# Scrub Typhus in Children at a Tertiary Hospital in North India: Clinical Profile and Complications

**Published:** 2014-07-19

**Authors:** Nowneet Kumar Bhat, Minakshi Dhar, Garima Mittal, Nadia Shirazi, Anil Rawat, Bram Prakash Kalra, Vipan Chandar, Sohaib Ahmad

**Affiliations:** 1 Department of Pediartics; 2 Department of Medicine; 3 Department of Microbiology; 4 Department of Pathology, Himaliyan Institute of Medical Sciences, Swami Rama Himalayan University, Doiwala, Dehradun, Uttarakhand, India

**Keywords:** Scrub Typhus, Mite, Eschar, Rash, Meningoencephalitis

## Abstract

***Objective:*** To study the clinical profile and complications of childhood scrub typhus.

***Methods:*** Prospective observational study of 66 children with scrub typhus, admitted to a tertiary hospital in north India, during the period between January 2011 and December 2012. The diagnosis was confirmed by serology.

***Findings*** ***:*** All children presented with fever. Other common symptoms were vomiting (56%), facial swelling (52%), cough (35%), abdominal pain (33%), breathlessness (29%) and decreased urine output (29%). High grade fever (>101^ o^F) was recorded in 91% of children. Other common signs were hepatomegaly, splenomegaly, edema, tender lymphadenopathy and hypotension, observed in 82%, 59%, 39%, 38% and 36% of cases, respectively. An eschar and a maculopapular rash each were observed in 20% of patients. Meningoencephalitis (30.3%), severe thrombocytopenia (27.2%), shock (25.8%), acute kidney injury (16.7%) and hepatitis (13.6%) were the most common complications observed in these children. Other common complications were acute respiratory distress syndrome, respiratory failure requiring ventilation, bronchopneumonia and myocarditis. Ninety percent of children became afebrile within 48 hours of initiating an appropriate antibiotic. Median time to defervescence was 22 hours. The overall mortality rate was 7.5%. Causes of death were refractory shock, meningoencephalitis, acute respiratory distress syndrome, bronchopneumonia, acute kidney injury and myocarditis.

***Conclusion:*** Pediatricians should keep a high index of suspicion for scrub typhus in any febrile child having a maculopapular rash, hepatosplenomegaly, tender lymphadenopathy, thrombocytopenia and features suggestive of capillary leak. Pending serological confirmation, empirical therapy with doxycycline or azithromycin should be started, as delay in treatment would result in life threatening complications.

## Introduction

Scrub typhus is an important cause of acute undifferentiated fever in rural Asia, northern Australia, and the western Pacific islands^[^^1^^]^. The majority of studies regarding rickettsial infections from various parts of the world are based on adult populations^[^^2^^-^^4^^]^. There is a paucity of studies regarding the incidence and clinical profile of scrub typhus in children^[^^5^^-^^12^^]^, despite epidemiological mention of children constituting up to half of scrub typhus cases in some regions. The majority of published studies are retrospective^[^^5^^,^^6^^,^^8^^,^^9^^]^ or sporadic case reports^[^^10^^-^^12^^]^. Scrub typhus, is now the most commonly reported rickettsial infection from the Indian subcontinent^[^^4^^,^^5^^,^^9^^,^^13^^-^^16^^]^. Scrub typhus and other rickettsial infections are grossly under-diagnosed in India because of their non-specific clinical presentation, low index of suspicion among clinicians, limited awareness about the disease and lack of diagnostic facilities^[^^4^^]^.

 We conducted a prospective observational study at a teaching hospital in north India, to study the clinical features and therapeutic outcomes of pediatric scrub typhus. The research objective was to determine the profile of children presenting with scrub typhus at our institution and whether it is different from that reported previously.

## Subjects and Methods

The study was conducted in the Department of Pediatrics, Himalayan institute of Medical sciences, SRH University, a tertiary care teaching hospital at Dehradun, India over a period of 2 years from January 2011 to December 2012. The hospital caters to patients from Uttarakhand, western Uttar-Pradesh and other adjoining areas of sub Himalayan north India. Scrub typhus was suspected in all children up to 18 years of age who had a fever for more than 5 days without an identifiable infection and one or more of the following clinical features: rash, edema, hepatosplenomegaly, lymphadenopathy and an eschar^[^^6^^]^. Serological diagnosis was made by a rapid immunochromatographic assay (SD Bioline Tsutsugamushi test from Standard Diagnostics, Inc. Hagal-dong, Kyonggi-do, Korea) and/or IgM ELISA test (Scrub typhus detect^TM^ IgM ELISA system from In BiOS International, Inc. Seattle USA). A favorable clinical response to antibiotics (defervescence within 48 h) was considered additional evidence of the disease^[^^2^^,^^15^^-^^17^^]^.

 Clinical data, including the duration of fever, associated symptoms, vital signs, and the general and systemic examination findings, were recorded. Patients were treated with a 10-day course of antibiotics (doxycycline 4 mg/kg/day BD or chloramphenicol 100 mg/kg/day qid). The response to treatment, the defervescence time, and the complications were noted. A careful search for eschar was performed in all patients. Data regarding age, sex, residential area, exposure to animals, exposure to farming and proximity to bushy and forest areas were collected.

 Complete blood counts, chest X-rays, tests for renal and liver function, urinalysis and serum electrolyte estimation were performed at presentation for all cases and were repeated if necessary. Common infectious conditions that could clinically mimic scrub typhus were ruled out by performing the following tests: peripheral smear and rapid antigen test for malaria, Widal test, Dengue(NS1 antigen and IgM antibody) test, urine and blood cultures. Tuberculin test, leptospira serology and an HIV-ELISA were performed when clinically indicated. Cardiac evaluation and cerebrospinal fluid (CSF) analysis was performed for selected cases with suspected myocarditis or meningoencephalitis respectively. The data was entered into a Microsoft Office excel spreadsheet and analyzed.

## Findings

Sixty six children (39:M, 27:F) were diagnosed with scrub typhus during the study period with a male to female ratio of 1.44:1. All cases were serologically confirmed. The age of the patients ranged from 8 months to 18 years with a mean age of 8.8 years. Two thirds of all children were <12 years of age with children between 12 and 18 years of age accounting for remaining one third of cases. Fifty five (83.3%) cases were observed between the months of September and November. Forty one (62%) cases were from the hilly Garhwal division of Uttarakhand whereas 25 (38%) cases belonged to adjoining non-hilly Saharanpur and Bijnor districts of western Uttar-Pradesh. Overall 58 (88%) cases resided in rural areas. Various environmental risk factors, such as living close to forests, bushes or crop fields, were present in 55 (83.3%) patients. A history of exposure to domestic animals (cattle, dogs) was found in 62 (93.9%) patients. 

**Table 1:** Clinical profile (signs and symptoms) of children with scrub typhus at presentation

**Symptoms**	**No. (%)**	**Signs**	**No. (%)**
**Fever **	66 (100)	**Fever >101** ^o^ **F**	60 (91)
** <7 days**	18 (27)	**Tachypnea**	21 (32)
** 7-14 days**	39 (59)	**Hypotension**	24 (36)
** >14 days**	09 (14)	**Eschar**	13 (20)
**Vomiting**	37 (56)	**Maculopapular rash**	13 (20)
**Cough**	23 (35)	**Lymphadenopathy**	25 (38)
**Headache**	12 (18)	**Hepatomegaly**	54 (82)
**Myalgia**	09 (14)	**Splenomegaly**	39 (59)
**Abdominal pain**	22 (33)	**Edema**	26 (39)
**Jaundice**	06 (9)	**Ascites**	10 (15)
**Diarrhea**	04 (6)	**Crackles/wheeze**	13 (20)
**Swelling** a	34 (52)	**Elevated JVP**	5 (8)
**Breathlessness**	19 (29)	**Meningeal signs**	12 (18)
**Seizures**	13 (20)	**Altered sensorium**	15 (23)
**Bleeding **	08 (12)	**Cranial nerve palsy**	02 (3)
**Oliguria**	19 (29)	**Petechiae/purpura**	06 (9)

a Includes swelling over any part of the body (e.g. facial, leg or generalized swelling)

**Fig. 1:**
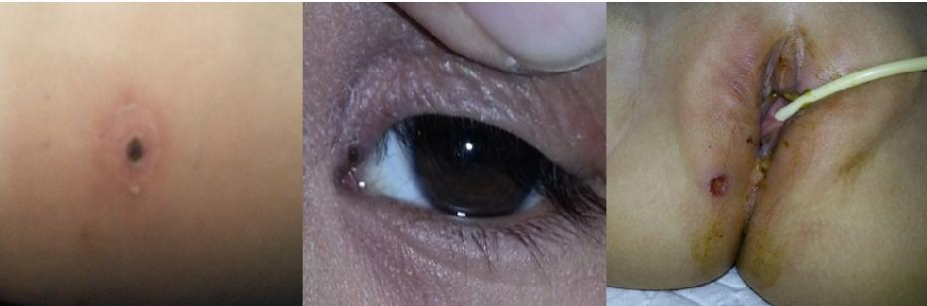
Typical eschar of scrub typhus

The clinical features at the time of presentation are shown in Table 1. All 66 patients presented with fever. The duration of fever on presentation ranged from 2 to 25 days with a median of 8 days. Other common symptoms were vomiting (56%), swelling especially facial (52%), cough (35%), abdominal pain (33%), breathlessness (29%) and decreased urine output (29%). High grade fever (>101^o^F) was recorded in 60 (91%) children. Other common signs hepatomegaly, splenomegaly, edema, tender lymphadenopathy and hypotension were observed in 82%, 59%, 39%, 38% and 36% of cases, respectively. An eschar (Fig 1) and a maculopapular rash each were observed in 20% of patients. Groin and axilla were the most common sites of eschar (70%). Anemia (hemoglobin <11.0 g%) was present in 41 (62%), thrombocytopenia (platelet count <1,00,000/mm^3^) in 35 (53%) and elevated liver enzymes (SGOT, SGPT) in 34 (51%) children. Severe anemia (hemoglobin <6.0 g%) was present in 4 (6.1%) children and severe thrombocytopenia (platelet count <50,000/mm^3^) in 18 (27.2%). Meningoencephalitis was the most common complication seen in 20 (30.3%) children. Lumbar puncture in all these children showed mononuclear pleocytosis. Seventeen (25.8%) children presented with shock and 8 (12%) children with respiratory failure needed assisted ventilation. Other complications encountered in the present study were pneumonia, pleural effusion, pericardial effusion, acute kidney injury (AKI), hepatitis, acute respiratory distress syndrome (ARDS) and disseminated intravascular coagulation (Table 2). 

**Table 2:** Complications of scrub typhus seen in the present study

**Complications **	**No.**	**%**
**Meningoencephalitis **	20	30.3
**Platlet count <50,000/mm** ^3^	18	27.2
**Shock **	17	25.8
**Acute kidney injury(AKI)**	11	16.7
**Hepatitis **	09	13.6
**Acute respiratory distress syndrome(ARDS) **	08	12.1
**Respiratory failure**	08	12.1
**Pneumonia **	07	10.6
**Cardiac dysfunction (myocarditis) **	06	9.1
**Pleural effusion**	06	9.1
**Severe anaemia (Hb <6gm%)**	04	6.1
**Pericardial effusion**	03	4.5
**Disseminated intravascular coagulation(DIC) **	01	1.5

 Doxycycline was used for treatment in 46 (69%) children. Parentral chloramphenicol was given to remaining children, who were seriously ill or not able to tolerate oral feeds. Sixty (90%) children became afebrile within 48 hours of initiating antibiotics which were continued for 10 days. The median time to defervescence was 22 h (range 12 to 60 h). Five children died with mortality rate of 7.5%. All these children presented with refractory shock. Other contributors to mortality were meningo-encephalitis (60%), ARDS (40%), broncho-pneumonia (40%), AKI (40%) and myocarditis (20%).

## Discussion

Scrub typhus is an acute febrile illness caused by rickettsia *Orientia tsutsugamushi*. The disease is transmitted to humans through the bite of an infected chigger, the larval stage of trombiculid mite^[^^18^^]^. The bacteria multiply at the inoculation site with the formation of a papule that ulcerates and becomes necrotic, evolving into an eschar, with regional lymphadenopathy that may progress to generalized lymphadenopathy within few days. Vasculitis is the basic pathogenic mechanism in scrub typhus. It is responsible for skin rash, microvascular leakage, edema, tissue hypo-perfusion and end organ ischemic injury^[^^19^^]^.

 In this prospective study, we describe the profile of pediatric scrub typhus at a tertiary hospital in northern India. There were more male patients than female patients, and the male-to-female ratio was 1.44:1, which is probably due to higher prevalence of exposure to chiggers among boys, who like to play outdoors^[^^4^^,^^6^^,^^8^^,^^13^^-^^17^^,^^20^^]^. The mean age at presentation was 8.8 years, which is similar to that reported by other authors^[^^13^^,^^17^^]^. The majority of cases occurred between the months of September and November, which follow the rainy monsoon season and coincide with the peak growth of vegetations and mite population. Similar observations have been recorded by other authors^[^^4^^,^^7^^,^^9^^,^^10^^,^^13^^,^^15^^,^^16^^,^^20^^]^, whereas a study from Taiwan found the greatest number of cases between May and August^[^^17^^]^.

 The clinical manifestations of scrub typhus in children are nonspecific and likely to be misdiagnosed. Fever was documented in all children in the present study similar to observations by other authors^[^^13^^-^^15^^]^. Features of capillary leak usually accompany fever, which is an important clinical finding to differentiate it from dengue fever. Twenty percent of patients had a maculopapular rash similar to that reported in a previous study^[^^13^^]^. Others have reported a higher occurrence of rash ranging from 23% to 100^%[^^9^^,^^21^^]^. The presence of an eschar is a valuable clinical clue in the diagnosis of scrub typhus; however absence does not rule out the disease. Eschar is a black necrotic lesion resembling a cigarette burn, usually found in areas where skin is thin, moist or wrinkled and where the clothing is tight like axilla, genitalia and inguinal area. Eschar was seen in 20% children in the present study, similar to that of some earlier reports^[^^7^^,^^13^^]^. In contrast, some authors have reported eschar in 50-80% of cases^[^^3^^,^^4^^,^^14^^,^^16^^,^^17^^]^. Others did not find an eschar in any of their cases^[^^5^^,^^8^^-^^11^^,^^20^^]^. Puffiness of face and pedal edema were observed in 52% and 39% respectively in the present study, compared with 63% and 60% reported in a previous study^[^^13^^]^. Vomiting (56%), cough (35%), abdominal pain (33%), breathlessness (29%) and seizures (20%) were the other common symptoms in the present study. A study from south India has reported these symptoms in 49%, 51%, 34%, 13% and 11% respectively^[^^13^^]^. Huang et al from Taiwan reported cough in 50% and vomiting in 29% of cases^[^^17^^]^. We observed hepatomegaly and splenomegaly in 82% and 59% of cases, respectively, whereas other authors have reported hepatomegaly in 59% to 98% and splenomegaly in 18% to 88% respectively^[^^13^^,^^14^^,^^22^^]^. The presence of splenomegaly is an important sign to distinguish scrub typhus from dengue fever, since splenomegaly is uncommon in the latter. Tender lymphadenopathy was observed in 38% of cases in the present study. Other authors have reported lymphadenopathy in 18% to 62% without mentioning about tenderness^[^^9^^,^^13^^,^^14^^,^^22^^]^. Thrombocytopenia was the major laboratory finding observed in the present study which was encountered in 53% with a reported frequency in literature of 22% to 78%^[^^13^^,^^14^^,^^16^^,^^22^^]^. Thrombocytopenia though a major finding was not associated with an elevated hematocrit, a valuable clue which helps to differentiate scrub typhus from dengue fever.

 Scrub typhus is regarded as a life threatening disease in children. Serious complications of scrub typhus usually occur in the second week of illness, which include ARDS, pneumonia, meningoencephalitis, AKI, myocarditis, severe thrombocytopenia and bleeding. Meningoencephalitis in 30.3% of cases, was the most common complication observed in the present study, whereas others have reported it in 5% to 19%^[^^6^^,^^13^^,^^14^^]^. Hypotension requiring ionotropic support (shock) was observed in 25.8% of cases against 45% reported in a previous study^[^^14^^]^. Another common complication was AKI, which was found in 16.7% of cases. Rickettsial infections have often been overlooked as a cause of AKI, especially in children. A recent retrospective study from central India did not report any case of AKI in children with rickettsial infections^[^^6^^]^. Three previous studies based on pediatric scrub typhus have reported lower incidences of AKI ranging from 2 to 10%^[^^9^^,^^14^^,^^23^^]^, whereas another study from south India has reported a higher frequency of 20%^[^^13^^]^. In adult studies, AKI has been described in 12-22% of cases^[^^3^^,^^4^^]^. AKI that is caused by acute tubular necrosis is a result of direct invasion by *Orientia tsutsugamushi*^[^^24^^]^.

 Pneumonia was observed in 10.6% of cases in the present study, while others have reported pneumonia in 3% to 21% of cases^[^^3^^,^^9^^,^^13^^,^^14^^]^. ARDS and myocarditis were reported in 12% and 9% of cases, respectively in the present study. A study from south India has reported a high frequency (34%) of myocarditis^[^^13^^]^. ARDS in literature has been reported in 4% to 22% of cases^[^^3^^,^^13^^,^^14^^]^. Twelve percent of children in present study as opposed to 35% in a study from south India^[^^14^^]^ developed respiratory failure and needed assisted ventilation.

 The high incidence of shock, acute kidney injury and myocarditis observed in the present study have diagnostic and therapeutic implications. Many clinical features including fever, hepatomegaly, edema, hypotension, thrombocyto-penia, and hepatitis can also be caused by dengue infection, which results in diagnostic confusion. The presence of other indicators such as an eschar, tender lymphadenopathy, splenomegaly, persistence of fever after the shock has supervened and the absence of an increase in hematocrit helps distinguish rickettsial infection from other hemorrhagic fevers, such as dengue^[^^13^^,^^14^^,^^22^^]^. Most of the patients in this study demonstrated a remarkable clinical response to doxycycline or chloramphenicol, as in other studies^[^^8^^,^^9^^,^^13^^-^^17^^,^^20^^]^. This dramatic response has also been used as a diagnostic test^[^^4^^,^^15^^-^^17^^]^.

 Most (90%) of the patients became afebrile within 48 h (median 22h). Mortality rate in the present study was 7.5% which is less than 15%, 12% and 9% reported by other authors^[^^6^^,^^14^^,^^20^^]^.

 The present study has some limitations. First the study was performed at a tertiary referral hospital; therefore does not reflect the actual burden of scrub typhus in the community, which may be higher. Second, rapid immunochromato-graphic assay and IgM ELISA were used for serological diagnosis because the indirect immunofluorescence assay, the gold standard confirmatory test is not yet widely available in India.

## Conclusion

When a child presents with acute febrile illness, maculopapular rash, hepatosplenomegaly, tender lymphadenopathy, thrombocytopenia and features suggestive of capillary leak, diagnosis of scrub typhus must be suspected and an eschar, if found is very useful for diagnosis. Clinical suspicion of scrub typhus warrants immediate empirical therapy with doxycycline or azithromycin pending serological confirmation, as delay in treatment would result in life threatening complications. Timely recognition of complications is of paramount importance to ensure a favorable outcome.

## References

[1] Silpapojakul, K (1997). Scrub typhus in the Western Pacific Region. Ann Acad Med Singapore.

[2] Panpanich R, Garner P (2002). Antibiotics for treating scrub typhus. Cochrane database Syst Rev.

[3] Lee CS, Min IS, Hwang JH (2010). Clinical significance of hypoalbuminemia in outcome of patients with scrub typhus. BMC Infect Dis.

[4] Vivekanandan M, Mani A, Priya YS (2010). Outbreak of scrub typhus in Pondicherry. J Assoc Physicians India.

[5] Mahajan SK, Rolain JM, Sankhyan N (2008). Pediatric scrub typhus in Indian Himalayas. Indian J Pediatr.

[6] Rathi NB, Rathi AN, Goodman MH (2011). Rickettsial diseases in Central India: proposed clinical scoring system for early detection of spotted fever. Indian Pediatr.

[7] Somashekar HR, Moses PD, Pavithran S (2006). Magnitude and features of scrub typhus and spotted fever in children in India. J Trop Pediatr.

[8] Murali N, Pillai S, Cherian T (2001). Rickettsial infection in south India - how to spot the spotted fever. Indian Pediatr.

[9] Digra SK, Saini GS, Singh V (2010). Scrub typhus in children: Jammu experience. JK Science.

[10] Pavithran S, Mathai E, Moses PD (2004). Scrub typhus. Indian Pediatr.

[11] Joshi R, Punde A, Ohri A (2009). Rickettsial infections seen in rural India. Bombay Hosp J.

[12] Somu S, Desingh SK (2010). The eschar of scrub typhus. Indian J Pediatr.

[13] Kumar M, Krishnamurthy S, Delhikumar CG (2012). Scrub typhus in children at a tertiary hospital in southern India: Clinical profile and complications. J Infect Public Health.

[14] Palanivel S, Nedunchelian K, Poovazhagi V (2012). Clinical Profile of Scrub Typhus in Children. Indian J Pediatr.

[15] Sirisanthana V, Puthanakit T, Sirisanthana T (2003). Epidemiologic, clinical and laboratory features of scrub typhus in thirty Thai children. Pediatr Infect Dis J.

[16] Chanta C, Chanta S (2005). Clinical study of 20 children with scrub typhus at Chiang Rai Regional Hospital. J Med Assoc Thai.

[17] Huang CT, Chi H, Lee HC (2009). Scrub typhus in children in a teaching hospital in eastern Taiwan, 2000-2005. Southeast Asian J Trop Med Public Health.

[18] Reller ME, Dumler JS, Kleigman RM (2011). Scrub Typhus (Orientia tsutsugamushi). Nelson Textbook of Pediatrics.

[19] Rathi N, Rathi A (2010). Rickettsial infections: Indian perspective. Indian Pediatr.

[20] Kamarasu K, Malathi M, Rajagopal V (2007). Serological evidence for wide distribution of spotted fevers & typhus fever in Tamil Nadu. Indian J Med Res.

[21] Mathai E, Lloyd G, Cherian T (2001). Serological evidence of continued presence of human rickettsiosis in southern India. Ann Trop Med Parasitol.

[22] Silpapojakula K, Varachita B, Silpapojakulb K (2004). Paediatric scrub typhus in Thailand: a study of 73 confirmed cases. Trans R Soc Trop Med Hyg.

[23] Kulkarni A, Vaidya S, Kulkarni P (2009). Rickettsial disease-an experience. Pediatr Infect Dis.

[24] Kim DM, Kang DW, Kim JO (2008). Acute renal failure due to acute tubular necrosis caused by direct invasion of Orientia tsutsugamushi. J Clin Microbiol.

